# Stressful life events as precipitants of obsessive–compulsive disorder: a systematic review and meta-analysis

**DOI:** 10.1017/S1092852925100497

**Published:** 2025-09-19

**Authors:** Veronica Hühne, Samara dos Santos-Ribeiro, Maria Eduarda Moreira-de-Oliveira, Gabriela B. de Menezes, Leonardo F. Fontenelle

**Affiliations:** 1 Anxiety, Obsessions and Compulsions Program, Institute of Psychiatry of the Federal University of Rio de Janeiro (UFRJ), Rio de Janeiro, Brazil; 2 Anxiety and Obsessive-Compulsive Disorders Research Unit, D’Or Institute for Research and Education (IDOR), Rio de Janeiro, Brazil; 3 Department of Psychiatry, School of Clinical Sciences, MONASH University, VIC, Australia

**Keywords:** Obsessive–compulsive disorder, stressful life events, risk factors, OCD onset, systematic review

## Abstract

**Objectives:**

Much work has been done on the role of trauma in obsessive–compulsive disorder (OCD), but the relationship between stressful life events (SLEs) and the onset of OCD remains poorly studied. This study aims to summarize the evidence about the association between SLEs and OCD development.

**Methods:**

For this systematic review, we searched PubMed, Web of Science, Scopus, and PsycINFO databases for studies published from the database’s inception to December 12, 2024. We included studies investigating the prevalence of SLEs among individuals diagnosed with OCD compared to other psychiatric disorders or healthy controls.

**Results:**

Seven studies met the inclusion criteria and were incorporated. Two studies found that OCD patients suffered more SLEs than healthy controls in the year before the onset of OCD. Two of the included studies showed a higher occurrence of SLEs across the patients’ lifetime before the onset of OCD. Three studies were comparable and, therefore, meta-analyzable. Together, they revealed that SLEs in the year before the onset of OCD were associated with a small yet positive pooled effect size.

**Conclusions:**

Our review suggests that SLEs may be highly represented among people with OCD both in the year preceding the disorder’s onset and throughout their lifetime.

## Introduction

Obsessive–compulsive disorder (OCD) is characterized by unwanted and intrusive thoughts, urges, or images that cause anxiety or distress (obsessions) and repetitive mental or motor acts (compulsions).^1^ OCD is relatively common, with a global prevalence of approximately 3%.^2^ Although there is consistent evidence showing that OCD has a significant genetic component,^3^ it has also been speculated that lifelong experiences, such as perinatal events, infections, exposure to toxic pathogens, and traumatic and stressful life events (SLEs)^4^ can alter the manifestation of OCD-related genes through epigenetic mechanisms.^5^ Recently, interest in the role of traumatic events in OCD, both in childhood^6^ and during adult^7^ years, has significantly grown.

Trauma and SLEs are related but distinct concepts. The former is the experience of actual or threatened death, serious injury, or sexual violence.^1^ The latter does not have a clear definition in the literature but is probably broader for including both traumatic and nontraumatic events that could otherwise induce stress and require an adaptative period after their occurrence.^8^ ^,^^9^ A study found that SLEs perceived as traumatic were associated with clinical levels of posttraumatic stress symptoms, depression, and low quality of life (QoL).^10^ The role of SLEs in the onset, severity, and treatment response of different mental disorders has been investigated over the past six decades.^11^ ^,^^12^

In these studies, SLEs were assessed through self-report tools and semistructured interviews, such as the *Life Event Checklist^13^* and the *Paykel Life Events Scale*,^14^ respectively. The use of formal tools to investigate SLEs may be desirable for actively prompting participants to recall SLEs by asking about various specific situations that occur during one’s lifetime (eg, change in schools) rather than asking in general and relying solely on the participants’ interpretation of what consists a SLE. Instruments that access SLEs encompass not only undesired events, such as death or illness, but also desired events that can be stressful, like marriage or parenthood.^15^ This broader scope ensures that the impact of various life experiences is captured and considered.

Although most studies have investigated the association between SLEs and unipolar depression,^16^ ^–^^18^ there has also been interest in the role of SLEs in bipolar disorder,^19^ psychosis,^20^ and borderline personality disorder (BPD).^21^ Overall, these investigations suggest that SLEs play a role in the onset of these disorders.^11^ On the other hand, the relationship between SLEs and OCD has not been as thoroughly studied. Although it is intuitive to assume that SLEs lead to worse clinical outcomes in OCD samples, earlier observations indicated that certain events (eg, joining the military) may actually also lead to remission.^22^ Understanding whether SLEs are a risk or a protective factor for OCD is crucial so that clinicians can be vigilant and potentially screen for obsessive–compulsive symptoms (OCS) following a SLE.

Extensive research has delved into the intricate connection between trauma and OCD.^7^ ^,^^23^ ^,^^24^ These studies have sought to uncover various aspects of this relationship, such as whether traumatic events serve as triggers for the onset of OCD,^25^ ^,^^26^ whether specific types of trauma exhibit a stronger association with OCD onset,^27^ their potential correlation with specific domains of OCS,^28^ their association with increased symptom severity,^27^ ^,^^29^ and even their role in rendering OCD treatment resistant.^24^ A distinct subtype of OCD, characterized by its association with trauma, has been proposed.^30^ However, in contrast to the work performed on the role of trauma in OCD, the correlation between SLEs and OCD remains relatively scarce.

This study aims to summarize the existing evidence concerning the association between SLEs and the development of OCD. This systematic review sought to answer the following question: Do individuals diagnosed with OCD experience a different frequency of SLEs from the one shown by individuals with other psychiatric disorders and healthy controls?

## Methods

This review was registered at the International Prospective Register of Systematic Reviews (PROSPERO) under the number [CRD42020206894].^31^ The reporting of this systematic review was guided by the standards of the Preferred Reporting Items for Systematic Review and Meta-Analysis (PRISMA) Statement.^32^

### Criteria for considering studies for this review

#### Types of studies

We searched for case–control studies.

#### Types of participants

The target population was people diagnosed with OCD using recognized diagnostic criteria assessed by a formal/valid diagnostic tool.

#### Types of comparators

We compared OCD patients with people not diagnosed with OCD (i.e., people diagnosed with other psychiatric disorders or not diagnosed with any psychiatric disorder [healthy controls]). The diagnosis of other conditions also had to be made using recognized diagnostic criteria.

#### Types of outcome measures

To be included, a study had to evaluate the presence of SLEs with formal/valid tools.

### Search methods for identification of studies

The search was done on December 12, 2024, in the following databases: PubMed, Web of Science, PsycINFO, and Scopus. No date constraint was adopted, but only studies in English were included. In addition, one independent reviewer (M.M.) performed a hand search of the selected studies’ reference lists to supplement the database search. The search strategy for each database is presented in Table 1.

**Table 1.** Online Search Features

Database	Field of search	Search strategies
PubMed	Any field	(Obsessive–compulsive disorder OR OCD) AND (stressful life event* OR recent life event*)
PsycINFO		
Web of Science	All fields	
Scopus	Article, title, and keywords	

Duplicate titles across databases were removed. Two pairs of independent reviewers, either V.H. and S.S. or V.H. and M.M. assessed the articles. The screening was performed in two phases: first, assessing the title and abstract and then examining the full text. Articles that did not meet the inclusion criteria were excluded. In case of disagreements, the three researchers (V.H., M.M., and S.S.) discussed the matter to reach a consensus. If consensus could not be reached, a fourth researcher, either L.F. or G.M., made the decision. Unavailable reports were requested from the authors via email.

### Data extraction and management

The data extraction was performed by one researcher (V.H.) and revised by two others (M.M. and S.S.). Any disagreement was resolved during our meetings. Qualitative and quantitative data were collected using an extraction template. We extracted information regarding the country of study, population, demographics, diagnostic method, disorder severity, control group, study design, and primary outcome data (SLE scale and measurement, like mean number of SLEs and their standard differences). Most of the studies included in this review investigated SLEs by applying the scale to the patient and their relatives. A double check was performed to prevent memory bias by confirming the event and its timeline with other sources. We have contacted the corresponding authors in case of doubts or the absence of critical information.

### Assessment of risk of bias in included studies

We assessed the risk of bias through the Joanna Briggs Institute (JBI) Critical Appraisal Checklist for Case–Control Studies.^33^ This checklist evaluates group comparability, the reliability of exposure and outcome assessments, the identification of confounding factors, and the adequacy of analysis. It includes questions such as: “Were the groups comparable other than the presence of disease in cases or the absence of disease in controls?” “Were outcomes assessed in a standard, valid and reliable way for cases and controls?” “Were confounding factors identified?” All papers were revised by two researchers, including V.H. and M.M. or S.S. Each aspect listed in the checklist was classified as “yes,” “no,” “unclear,” or “not applied” for each selected study. Our review only included the study if less than four aspects were classified as “no.” In case of doubts or insufficient information on the report, corresponding authors were contacted for clarification. In case of disagreements, a fourth researcher (either L.F. or G.M.) made the decision.

### Data analysis

All included studies were summarized with a qualitative synthesis, but three were able to be included in the meta-analysis. The meta-analysis data were plotted and managed in the software Comprehensive Meta-Analysis (CMA) version 4.^34^ The fixed effect analysis was chosen.

To quantify the effect size of each study, the mean and standard deviation (SD) of the *Paykel Life Events Scale* scores were utilized. The standard difference in means (SDM) was calculated as the effect size for each study. Based on the SDM values, the effect size was categorized as small for values between 0.2 and 0.5, medium for values between 0.5 and 0.8, and large for values greater than 0.8.^35^ To assess the heterogeneity among the included studies, the *I* ^2^ statistic was used and interpreted following the guidelines by Higgins.^36^ *I* ^2^ values falling within the ranges of 0–25%, 25–50%, and 50–75% indicate low, moderate, and high levels of heterogeneity, respectively. Additionally, the *Q* statistic was reported, assuming a *p* value of 0.05 as the threshold for significance for heterogeneity.

In the qualitative synthesis, when the *p* values for the difference between groups were available, we extracted them directly from the reports. For those who provided only mean values and their standard deviations, we calculated the difference using the *t* test for studies comparing two groups. The *p* values we calculated are signalized by ** at the table caption.

## Results

### Search results

**Figure 1.**
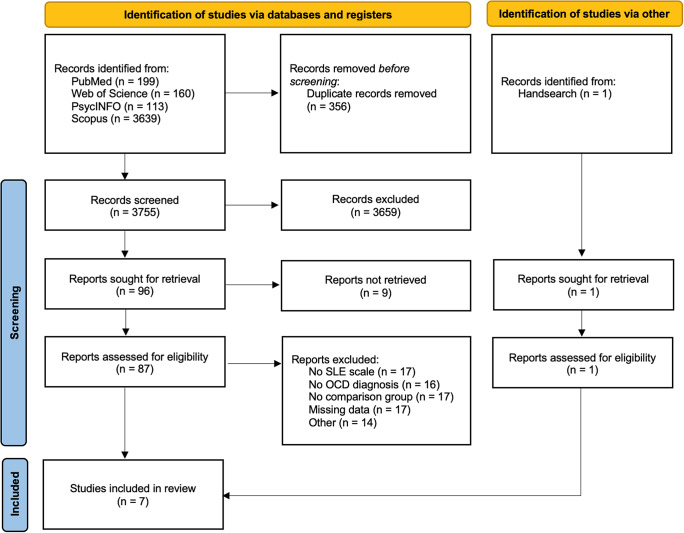
PRISMA flow diagram for systematic review.

The electronic search yielded 4111 records. Duplicate titles across databases were removed, leaving 3755 records to be screened. Seven were considered eligible according to the inclusion criteria mentioned earlier. Figure 1 shows the PRISMA flow chart with information about the stages of the search and screening process.

### Description of studies

A total of seven reports published between 1986 and 2014 were selected. These studies were conducted in various countries, including India (*n* = 3), Italy (*n* = 2), the Netherlands (*n* = 1), and England (*n* = 1). All studies had a cross-sectional case–control design.

The *Yale-Brown Obsessive–Compulsive Scale* (YBOCS)^37^ (*n* = 3) and the *Leyton Obsessional Inventory^38^* (*n* = 2) were used to assess the severity of OCD symptoms. SLEs were measured primarily not only with the *Paykel Life Events Scale* (*n* = 4),^14^ but also with the *Presumptive Stressful Life Event Scale* (*n* = 2)^39^ and the *Risky Family Questionnaire* (*n* = 1).^40^

The results are summarized in three tables, arranged by the SLE scale used: Table 2 presents the studies that employed the *Paykel Life Event Scale*, and Table 3 groups the studies that used other scales.

**Table 2.** Studies That Used the Paykel Life Event Scale

First author (year)	Study design	Diagnostic criteria		Group 1	Group 2	Statistics
McKeon et al. (1984)^41^	Cross-sectional	Research diagnostic criteria	Diagnosis	OCD	Healthy controls	*t* = N/A; *p* < 0.01
			*N*	25	25	
			Age (SD)	25	N/A	
			Gender (female)	N/A	N/A	
			Mean SLE (*n* of events ypo)	1.6	0.76	
Khanna et al. (1988)^42^	Cross-sectional	DSM-III and ICD–9 criteria	Diagnosis	OCD	Healthy controls	6 m: *t* = 3.44; *p* < 0.001 LT: *t* = 0.78; *p* > 0.05
			*N*	32	32	
			Age	27.13 (9.16)	25.4 (10.32)	
			Gender	25%	25%	
			Mean SLE (*n* of events ypo)	6 m: N/A LT: 1.66 (1.11)	6 m: N/A LT: 1.44 (1.12)	
			Severity scale	Leyton obsessional inventory	Leyton obsessional inventory	
			Mean severity (SD)	37.49 (11.4)	23.32 (38.96)	
de Loof et al. (1989)^43^	Cross-sectional	DSM-III criteria	Diagnosis	OCD	Panic disorder	*t* = 0.8747; *p* < 0.05
			*N*	15	25	
			Age	N/A	N/A	
			Gender (female)	N/A	N/A	
			Mean SLE (*n* of events ypo)	2.13 (1.1)	1.8 (1.2)	
Maina et al. (1999)^44^	Cross-sectional	SCID III	Diagnosis	OCD	Healthy controls	*t* = 0.993; *p* = 90.322
			*N*	68	68	
			Age	26.4 (9.4)	N/A	
			Gender (female)	48.5%	48.5%	
			Mean SLE (*n* of events ypo)	1.57 (1.31)	1.36 (1.15)	
			Severity scale	YBOCS	N/A	
			Mean severity (SD)	27.5 (6.4)	N/A	

*Note.* ypo = year prior to the disorder’s onset or interview (for healthy controls).

**Table 3.** Studies That Used Other Stressful Life Event Scales

First author (year)	SLE scale	Study design	Diagnostic criteria		Group 1	Group 2	Statistics
Kulhara et al. (1986)^45^	Presumptive stressful life events scale	Cross-sectional	ICD–9 criteria	Diagnosis	OCD	Healthy controls	*t* = N/A; *p* < 0.01
				*N*	24	24	
				Age (SD)	36.25 (5.85)	35.65 (4.95)	
				Gender (female)	41.6%	41.6%	
				Mean SLE (SD) (*n* of events ypo)	4.62 (1.03)	1.35 (0.57)	
				Severity scale	Leyton obsessional inventory	Leyton obsessional inventory	
				Severity (SD)	55.17 (9.92)	24.83 (6.32)	
Sarkhel et al. (2011)^46^	Presumptive stressful life events scale	Cross-sectional	ICD–10-DCR (OCD group) and GHQ5 (control group)	Diagnosis	OCD	Healthy controls	*t* = 3.95; *p* = 0.01 *t* = 5.53; *p* < 0.01
				*N*	10	10	
				Age (SD)	27.6 (3.89)	31.5 (5.64)	
				Gender (female)	30%	40%	
				Mean SLE (SD)	6 m: 7.3 (1.95) LT: 13 (3.56)	6 m: 4.4 (1.26) LT: 6.5 (1.08)	
				Severity scale	YBOCS	N/A	
				Severity (SD)	19.6 (4.4)	N/A	
Benedetti et al. (2014)^47^	Risky families questionnaire	Cross-sectional	SCID-I (DSM-IV)	Diagnosis	OCD	Healthy controls	*p* = 0.75**
				*N*	66	31	
				Age	N/A	N/A	
				Gender (female)	N/A	N/A	
				Mean ACE (SD)	25.23 (9.81)	25.9 (7.76)	
				Severity scale	YBOCS	N/A	
				Severity (SD)	N/A	N/A	

*Note.* 6 m = 6 months before the onset of OCD or before the interview (for healthy controls); LT = lifetime occurrence of SLE; ypo = year prior to the disorder’s onset or interview (for healthy controls); ACE = adverse childhood experience.

** Calculated results.

### Summary of the results of studies using the Paykel Life Events Scale

The Paykel Life Events Scale^14^ is a standardized instrument used to assess an individual’s exposure to SLEs over a given period. The studies included in this review used two versions of the scale: one listing 61 SLEs and another with 64 events, both encompassing personal, social, financial, occupational, and health-related SLEs. The scale does not distinguish between desirable and undesirable SLEs; its score is the sum of the number of reported SLEs. The included studies administered the scale through semistructured interviews to minimize recall bias. Unfortunately, it does not account for the *subjective perception* of stress associated with each event.

All included studies investigated SLE occurrence in the year before the disorder’s onset or the year before the interview. Other time frames, like 6 months before the disorder’s onset^41^ ^,^^42^ ^,^^44^ or the lifetime occurrence of SLE, were also investigated in some studies.^43^ Only the study by McKeon et al.^41^ identified a statistically significant difference in the occurrence of SLEs in the year prior to OCD onset compared to healthy controls (*p* = 0.01). Their findings revealed that OCD patients experienced a higher number of SLEs the year before OCD’s onset compared to the healthy control group.

Khanna et al.^42^ also compared the occurrence of SLEs in OCD patients and healthy controls. They found that the patient group experienced a higher number of SLEs 6 months prior to the onset of OCD (*p* < 0.001). de Loof et al.,^43^ on the other hand, did not observe a statistically significant difference in the mean occurrence of SLEs in the year prior to the onset of OCD compared to panic disorder. However, they found that patients with panic disorder experienced a higher rate of SLEs throughout the lifetime preceding the onset of the disorder than OCD patients (13.2 and 7.2 events, respectively; *p* < 0.01). Maina et al.^44^ reported that the mean occurrence of SLEs within a year or 6 months before the onset of OCD did not present a difference compared to healthy controls.

**Figure 2.**
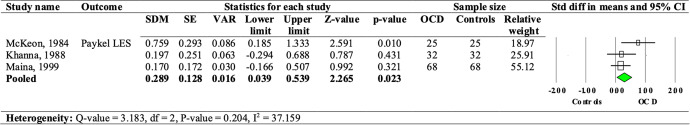
Forest plot of the mean difference of the Paykel Life Events Scale scores between the OCD group and the healthy controls group. Abbreviations: CI, confidence interval; LES, life events scale; OCD, obsessive–compulsive disorder; SDM, standardized difference in means; SE, standard error.

Since a reasonable number of studies compared OCD patients with healthy controls using the *Paykel Life Events Scale* (*n* = 3), a decision was made to combine their results and identify an overall effect size. To do so, data from 125 patients and 125 healthy controls were compiled and analyzed. The forest plot (Figure 2) illustrates the standard difference (in means) of the scores of the *Paykel Life Events Scale* between OCD patients and healthy controls in the year prior to OCD onset. The pooled effect size of SLEs in individuals with OCD was small and positive (SMD = 0.289, SE = 0.128, 95% CI: 0.039–0.539, *p* = 0.023), with a moderate heterogeneity across studies (*I* ^2^ = 37.159, *Q* = 3.183, *p* = 0.204).

### Summary of the studies using other stressful life event scales

Kulhara et al.^45^ found that the occurrence of SLEs in the year previous to OCD onset, especially undesired ones, was significantly more frequent in OCD patients than in healthy controls (4.62 and 1.35, respectively; *p* < 0.01). Aside from the total number of SLEs, the authors also looked at the desirability of these events. The OCD group had suffered more undesired SLEs than healthy subjects (mean 1.7 and 0.6, respectively; *p* < 0.01). Similarly, Sarkhel et al.^46^ found that OCD patients had suffered significantly more SLEs before the onset of the disorder than healthy subjects, both 6 months prior to the beginning (*p* = 0.01) or during their lifetime (*p* < 0.01). Benedetti et al.^47^ also investigated adverse childhood experiences (ACE) using the *Risky Family Questionnaire* (RFQ) in children diagnosed with OCD. Although the study focused on ACEs, the RFQ primarily assesses SLEs. The questionnaire includes items like, “How often did a parent or other adult in the household make you feel that you were loved, supported, and cared for?” and “Would you say that the household you grew up in was chaotic and disorganized?” The study compared RFQ scores between children with OCD and healthy controls, assessing ACEs that occurred one year before the onset of OCD or the year before the evaluation (in the control group). No statistically significant differences were found between the groups (*p* = 0.75).

### Results of the risk of bias assessment

All the selected articles were assessed for quality and risk of bias according to the JBI Critical Appraisal Checklist for Case–Control Studies.^33^ For the final evaluation of these studies by the JBI checklist, we also sought other sources for further information (supplementary material, articles listed in reference, and author contact). Supplementary Table S1 presents the assessment of these articles using the JBI checklist. No study was excluded from the review because of its risk of bias.

## Discussion

Our findings suggest that the relationship between SLEs and the onset of OCD is intricate and contingent upon the time frame examined. Our analysis revealed that SLEs occurring in the year prior to the onset of OCD, as well as in the lifetime of OCD participants before the onset of the disorder, were more commonly reported than in healthy controls, suggesting that SLEs may serve as a triggering factor on the development of the disorder. Therefore, it is essential to identify the time frame during which SLEs have the most influence on the onset of OCD to inform clinicians when to be vigilant and actively screen the appearance of OCS during that particular period.

Two studies included in our review found that individuals with OCD had experienced more SLEs during their lifetime prior to the disorder’s onset when compared to healthy subjects.^43^ ^,^^46^ Thus, we acknowledge that some SLEs (eg, early childhood events) may have a “delayed effect,” as some individuals may require a longer time to process these SLEs and only display OCD years after. Curiously, Gothelf et al.^48^ suggested that SLEs might influence one’s personality, causing it to be more susceptible to developing anxiety or other disorders. Similarly, Faravelli et al.^49^ found a connection between stress and hyperactivity of the hypothalamus–pituitary–adrenal (HPA) axis in individuals diagnosed with OCD. This HPA axis dysregulation could render the central nervous system vulnerable to OCD.^49^ It seems essential not only to investigate SLEs’ occurrence throughout an individual’s lifespan, but also closer to the disorder’s onset.

Most studies in this review focused solely on comparing individuals diagnosed with OCD to healthy controls, except for the study by de Loof et al.,^43^ which compared OCD with panic disorder. However, there remains a need for further research that compares the incidence of different types of SLEs across different psychiatric disorders. Such studies would contribute to a better understanding of how SLEs are associated with various disorders and whether they represent a common transdiagnostic risk factor. The absence of comparative analyses with other disorders leaves us uncertain about its broader applicability because comorbidities might be confounding factors for the association between SLEs and OCD onset.

Despite all included studies being cross-sectional case controls, we detected at least one prospective study that assessed the relationship between life events more broadly in OCD.^50^ The study by Valleni-Basile et al.^50^ reported that black race, age, fewer desirable and more undesirable life events, and socioeconomic status were significant predictors of incident OCD. Although this exclusion was based on methodological decisions taken in our planning phase, such as a focus on studies that compared OCD patients with individuals diagnosed with other psychiatric disorders or healthy controls, we believe there are some particularities of this study that make their results hard to interpret.

More specifically, Valleni-Basile et al.^50^ employed the *Coddington Life Events Scale for Adolescents*, among other measures, to investigate the relationship between the total number of “undesirable” and "desirable” life events occurring in the previous year and “incident” OCD after 1 year. The *Coddington Life Events Scale for Adolescents* is known to emphasize household dysfunction (eg, parents or guardians’ separation or divorce, violence between household members, and mental health or legal problems among the household members) as compared to situations outside of the home or of more traumatic abuse and neglect in relation to the child themselves.^51^ Thus, the somewhat restricted assessment of SLEs limits its comparability with other studies using more traditional and comprehensive assessment methods such as the *Paykel Life Events Scale.*

Traumatic events fall under the category of SLEs, though the inverse relationship is not true. Nevertheless, in our review, we chose not to include studies that compared the presence of traumatic events associated with the onset of OCD. This decision was guided by the abundance of studies exploring this relationship^26^ ^,^^52^ and the absence of reviews on the connection between SLEs and OCD. Hypothetically, a “traumatic event” is associated with greater psychological impact than a nontraumatic SLE. However, there is research showing that the subjective impact of an event can be as crucial as its objective severity.^10^ This recognition emphasizes that emotional and psychological experiences can be as impactful as the event’s objective characteristics to determine the potential impact on mental health outcomes.

Despite being more common occurrences in everyday life than traumatic events, SLEs have been poorly studied in OCD populations. New studies should be undertaken within clinical populations, encompassing a broader spectrum of SLEs, including those of a novel nature. The COVID-19 pandemic introduced a range of “new” stressors, such as social isolation, financial concerns, and heightened health anxieties.^53^ ^,^^54^ These stressors, distinct from those previously studied, may exert differing effects on the onset of psychiatric disorders.^55^ For many individuals with OCD, the pandemic has been a stressor, leading to symptom exacerbation or the emergence of new symptoms.^56^ Given the variability in protective measures and individual lifestyles, standardized evaluation instruments are necessary for cross-sample comparisons. Scales like the COVID Stress Scale (CSS)^57^ and the Coronavirus Traumatic and Stressful Life Events Scale (COROTRAS)^58^ have been developed to assess pandemic-related SLEs and have been used in a few studies.^59^ However, as none of these studies met the inclusion criteria for this review, they were excluded. Studies investigating these newly emerged events and their potential impact on the development of mental disorders, including OCD, become crucial to advise more nuanced clinical approaches.

Our review has significant limitations. First, several studies included in the review can be considered outdated as 10 years have passed since the last study that met the inclusion criteria was published, and the field has incorporated several sound instruments for the assessment of multiple SLEs in the previous few years.^51^ Second, the scales seem heterogeneous and hardly comparable (eg, the *Paykel Events Scale* vs. the *Presumptive Stressful Life Events Scale*, which is heavily culturally influenced). Third, most available scales assess only the presence or total number of SLEs rather than distinguishing specific SLE types (eg, desirable vs. undesirable). To address this gap, our group has developed a scale designed to assess adverse SLEs across various domains relevant to obsessive–compulsive and related disorders (OCRDs).^60^ In this scale, events were selected based on their perceived stressfulness for individuals on the OCRDs spectrum and an analysis of OCD symptom dimensions. Only SLEs that the subject perceives as significantly traumatic or stressful are recorded, as some may be considered neutral. This scale allows for (i) quantification of the total number of SLEs, (ii) categorization of SLEs by themes, and (iii) assessment of the emotional intensity associated with the most stressful event.

## Conclusion

The relationship between SLEs and the onset of OCD is complicated and time dependent. Our findings suggest the importance of actively investigating SLEs throughout a patient’s lifetime, particularly in the year before the manifestation of the disorder. We recommend that clinicians be vigilant of subclinical OCS in the aftermath of SLEs, as our review provides compelling evidence of OCD onset in the year following an undesired SLE. We suggest that future research expands beyond comparing the prevalence of SLEs solely between individuals diagnosed with OCD and healthy controls. It is equally critical to include comparisons with other patient groups, thereby elucidating whether SLEs represent an isolated risk factor specific to OCD or a broader phenomenon of transdiagnostic importance. Notably, several studies investigating SLEs are more than 30 years old, and the emergence of new stressors, particularly in the wake of the COVID-19 pandemic, has introduced novel challenges and experiences for patients and the general population alike. This necessitates a fresh wave of research to capture and analyze the impact of these new SLEs, thereby advancing our knowledge in this evolving field.

## Supporting information

Hühne et al. supplementary material Hühne et al. supplementary material

## Supplementary material

The supplementary material for this article can be found at http://doi.org/10.1017/S1092852925100497.
