# Supplementary material for: Stressful life events as precipitants of obsessive–compulsive disorder: a systematic review and meta-analysis
Source: CNS Spectr. 2025 Sep 19;30(1):e75. doi: 10.1017/S1092852925100497 (PMC13064784; doi:10.1017/S1092852925100497)
Supplement: Hühne et al. supplementary material [file S1092852925100497sup001.docx]

**Table S1: Assessment of selected studies according to the JBI Critical Appraisal Checklist for Case-Control Studies**

| **Checklist Item** | [1] | [2] | [3] | [4] | [5] | [6] | [7] |
| --- | --- | --- | --- | --- | --- | --- | --- |
| 1-Were the groups comparable other than the presence of disease in cases or the absence of disease in controls? | x | x |  | x | x | x |  |
| 2-Were cases and controls matched appropriately? | x | x |  | x | x |  | x |
| 3-Were the same criteria used for identification of cases and controls? |  |  | x | x |  |  |  |
| 4-Was exposure measured in a standard, valid and reliable way? | na | na | na | na | na | na | na |
| 5-Was exposure measured in the same way for cases and controls? | na | na | na | na | na | na | na |
| 6-Were confounding factors identified? |  |  |  |  |  |  |  |
| 7-Were strategies to deal with confounding factors stated? | na | na | na | na | na | na | na |
| 8-Were outcomes assessed in a standard, valid and reliable way for cases and controls? | x | x | x | x | x | x | x |
| 9-Was the exposure period of interest long enough to be meaningful? | na | na | na | na | na | na | na |
| 10-Was appropriate statistical analysis used? | x | x | x | x | x | x | x |

[1] = McKeon, J. (1984); [2] = Khanna (1988); [3] = de Loof, C. (1989); [4] = Maina, G. (1999)

[5] = Kulhara, P. (1986); [6] = Sarkhel, S. (2011); [7] = Benedetti, F. (2014)

x = item was assigned a yes; na = item was assigned as “not applicable”
